# Changes in Pain Perception following Psychotherapy: The Mediating Role of Psychological Components

**DOI:** 10.1155/2018/8713084

**Published:** 2018-04-19

**Authors:** Susanna Zanini, Alessandra Voltolini, Gaia Gragnano, Emilia Fumagalli, Francesco Pagnini

**Affiliations:** ^1^Clinical Psychology Service, ASST Grande Ospedale Metropolitano Niguarda, Piazza Dell'Ospedale Maggiore 3, 20162 Milan, Italy; ^2^Department of Psychology, Università Cattolica del Sacro Cuore, Largo Agostino Gemelli 1, 20123 Milan, Italy; ^3^Department of Psychology, Harvard University, 33 Kirkland Street, Cambridge, MA 02138, USA

## Abstract

Chronic pain is frequently associated with significant psychological issues, such as depression or anxiety. Psychological treatments, such as psychotherapy, can often alleviate both psychological and pain symptoms. However, there is limited research about the association between psychological symptoms and perceived pain in the context of psychotherapeutic interventions. We conducted a retrospective study that analyzed, in a hospital context, how changes in psychological functioning and well-being were associated with pain reduction. Thirty-seven records of patients with chronic pain attending psychotherapy in a public hospital were included. All patients were assessed before psychotherapy, as well as after 6 and 10 months, with self-reported questionnaires about pain, anxiety, depression, and psychological functioning. Results indicate that reductions in anxiety, depression, psychological problems, risk factors, and well-being are strongly related with a reduction in pain, further confirming the hypothesis that psychological morbidity is associated with pain severity.

## 1. Introduction

Pain is an unpleasant and distressing feeling that is often caused by an intense or damaging stimulus [1]. While pain is influenced by the stimulus intensity, it is also connected to cognitive and emotional factors, which can modulate the painful experience [2]. Therefore, similar painful stimuli may lead to different experiences among the individuals, on the base of contexts and psychological characteristics [3]. Considering the importance of the cognitive and emotional components in the experience of pain, psychological interventions, such as psychotherapy, could play an important role in pain rehabilitation and management [4].

Reactive psychological symptoms, such as depression and anxiety, may have an important role in the exacerbation of pain perception [5]. For example, de Heer and colleagues [6] have found, over a large sample of people with chronic pain, that depressive and anxiety disorders were associated with increased pain-related disability. This association with depression and anxiety remains stable over time [7, 8]. It has also been suggested that depression and pain may share similar neuroplastic changes in the central nervous system [9], which would explain the positive impact of antidepressants on the pain symptomatology [10]. Similarly, in the framework of a randomized controlled trial, the reduction of depression following an online cognitive-behavioral intervention was associated with pain decrease [11].

Psychosocial pain treatments, including psychotherapy, proved to be effective in reducing pain and promoting increased quality of life [12]. Most research, as it is often the case in clinical psychology, has been conducted in *true-experiment settings*, providing high internal validity for the results, but not necessarily generalizing to the actual clinical contexts. For this reason, we planned a study that analyses data from a hospital setting. We aimed at verifying whether the hypothesis of an association between psychological symptoms and perceived pain would be confirmed in the mainframe of psychotherapeutic interventions.

## 2. Materials and Methods

We conducted a retrospective cohort study based on medical records collected by the Service of Psychology at ASST Grande Ospedale Metropolitano Niguarda. Specifically, we selected the requests for pain management that involved a psychotherapeutic intervention. Medical records included psychological questionnaires about pain, anxiety, depression, and psychological morbidity.

We selected all the records that met the following criteria: outpatients with chronic, nononcological pain treated at Rheumatology, Pain Service, or Nephrology units at Niguarda Ca' Granda Hospital in Milan; who successfully attended a psychotherapeutic intervention with either Susanna Zanini or Alessandra Voltolini; with a defined chronic pain diagnosis for more than 3 months; and who were 18 or older. To access the service, patients provided informed consent about the fact that their data could be used for scientific analysis, resulting in publication only in aggregated forms.

All patients (*n*=37) were assessed before beginning psychotherapy (first ambulatory session) and after 6 and 10 months.

The records included the following scales:

- *QUID*: Italian pain questionnaire [13], which is a self-report instrument assessing pain characteristics. It includes a semantic interval scale consisting of 42 pain descriptors and is divided into four main classes: sensory, affective, evaluative, and mixed. The items can be combined together to provide a total pain score.
- *HADS*: Hospital Anxiety and Depression Scale [14] for the assessment of anxiety and depression in hospitalized patients. The scale does not include items that refer to physical aspects of anxiety and depression, and it is widely used in the hospital setting.
- *CORE-OM*: Clinical Outcomes in Routine Evaluation-Outcome Measure [15] for the assessment of psychological distress. It is a widely used scale with adequate reliability (*α* 0.75–0.95) developed to evaluate the outcome of psychological therapy. The questionnaire is composed of four subscales: functioning, problems, well-being, and risks. Item scores range from 0 to 4 (higher scores indicate more distress).

### 2.1. Interventions

The psychotherapeutic interventions were conducted by two experienced psychotherapists (Susanna Zanini; Alessandra Voltolini), either in individual or group settings, on the base of patients' needs. The average duration of the treatments was 10 months. The two psychotherapists were trained in cognitive-behavioral therapy (CBT; Alessandra Voltolini) [16] and short-term integrated therapy (Susanna Zanini) [17, 18].

CBT is generally based on the “A-B-C” (Antecedent-Belief-Consequence) model and aims at changing dysfunctional thoughts, emotions, and behaviors [19]. Some of the strategies promoted by CBT are problem solving, decision-making, scheduling, relaxation techniques, mindfulness training, role-playing, and others. The short-term, focus-based, integrated psychotherapy includes different therapeutic theories and techniques: a short psychoanalytic model, the cognitive-behavioral model, and the developmental model, which include life-cycle theories and findings from resilience researches [17]. The integration among different models allows the selection of the therapeutic strategy that best fit to every specific patient, according to his/her needs. Moreover, it allows to combine together techniques from different approaches or to use them sequentially [20].

### 2.2. Analysis

The mediation effect that resilience could exert on pain over the course of psychotherapy was explored with linear mixed models [21]. Time points were formatted as time variant to consider changes over time, and the random effect was used to account for intrasubject variability. We referred to an autoregressive (AR1) covariance matrix with heterogeneous variances across different assessments (this matrix provided better fit indexes than the “unstructured” covariance matrix) [22]. We followed the same procedure to analyze the effects of resilience on anxiety and depression. Linear mixed models were also used to explore repeated measures trends. Bonferroni correction was used to account for multiple analyses. Data were analyzed with SPSS software.

## 3. Results and Discussion

Table 1 reports the descriptive statistics of the sample, including demographic and clinical data.

**Figure 1:**
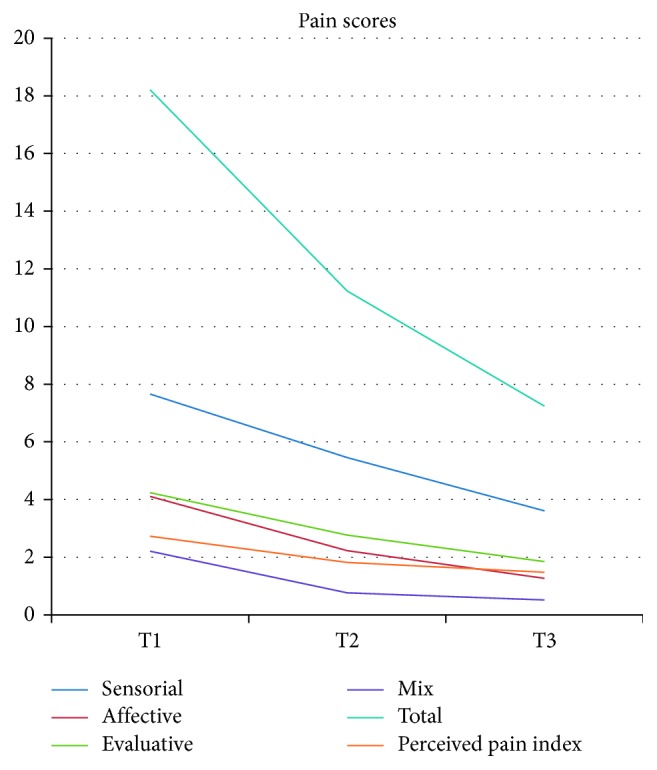
Time changes in pain scores.

**Figure 2:**
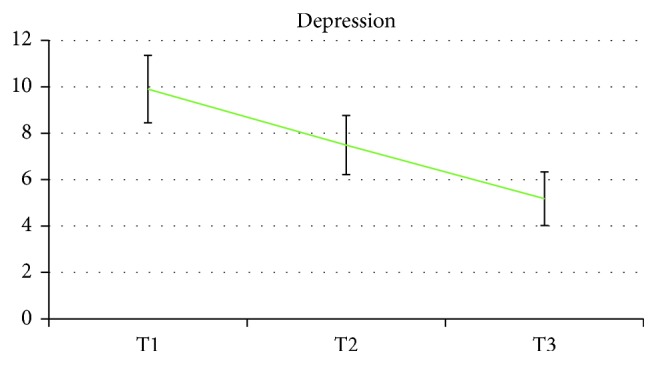
Time changes in depression scores.

**Figure 3:**
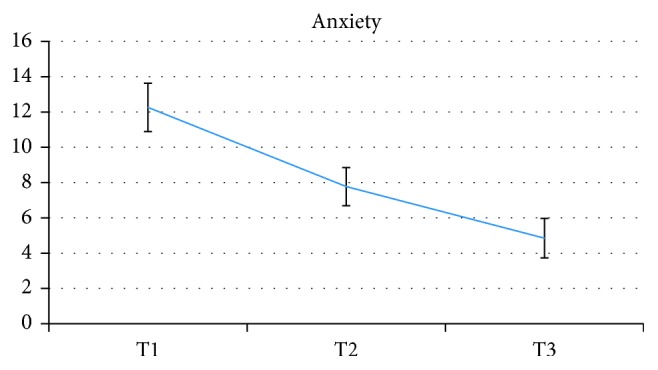
Time changes in anxiety scores.

**Figure 4:**
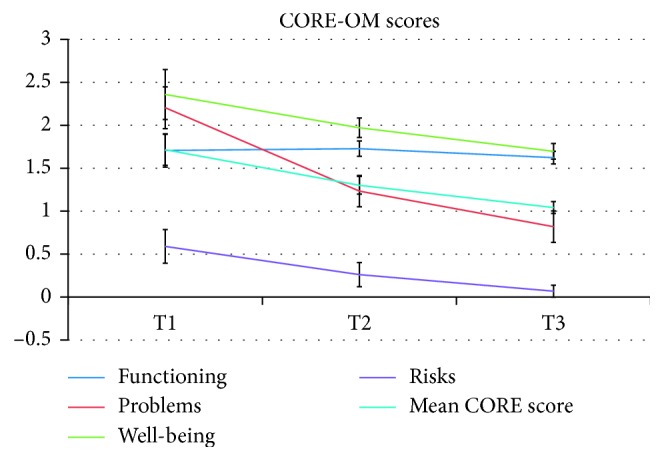
Time changes in the CORE-OM scores.

In time, there was a significant reducing trend among the three assessment times for pain and pain subscales (*p* < 0.001), anxiety (*p* < 0.001), and depression (*p* < 0.001). All CORE-OM scales showed significant reductions (*p* < 0.001 for all), with the only exception of functioning, which remained stable over time (*F*(2, 42.580) = 1.880, *p*=0.165). Time trends are reported in Figures 1–4.

Results from linear mixed models indicate that changes in both anxiety and depression strongly predicted total pain scores and specific pain components. All the subscales from the CORE-OM, with the exclusion of functioning, successfully predicted QUID scores, including both the total and the subscales. Beta scores, significance, and confidence intervals are detailed in Table 2.

As expected, we found strong correlations between psychological aspects and pain perceptions. Both pain and psychological morbidity significantly decreased over time. That is not surprising, as participants were all in psychotherapy and also treated for pain management in the hospital setting. The reduction of psychological symptoms, including anxiety and depression, resulted in association with pain reduction in all the assessed components. Interestingly, the scale functioning of the CORE-OM, which refer to general, interpersonal, and social functioning, did not result in association with pain. Accounting for this exception, our data draw a clear picture of strong associations between the psychological domains and the chronic pain experience. Results are in line with the previous research [23], and extend the association, sometimes found in controlled studies, in a natural setting (i.e., hospital).

The retrospective and observational nature of the study does not allow making a causal inference. In particular, the lack of a control group does not allow us to state that psychotherapy is what has brought to a reduction of the “negative” outcomes. From the perspective that we can honestly have with this study, we can observe and document the neat associations that anxiety, depression, psychological problems, risk factors, and well-being have with pain in all the assessed facets.

## 4. Conclusions

In a hospital setting, archives from outpatients with chronic pain attending psychotherapy and regular pain management were analyzed for associations between psychological factors and pain reduction. Results from this naturalistic setting indicate an association between psychological morbidity and pain severity.

## Figures and Tables

**Table 1:** Sample characteristics.

*N*	37
Sex (*N*, %)	Male: 13 (35.13%)
	Female: 24 (64.87%)
Age (mean, SD)	58.32 (13.26)
Disease (*N*, %)	Rheumatological conditions: 11 (29.72%)
	Nephrological conditions: 11 (29.72%)
	Nononcological chronic pain: 15 (40.54%)
Marital status (*N*, %)	Never married: 5 (13.51%)
	Married: 23 (62.16%)
	Divorced: 3 (8.1%)
	Widowed: 6 (16.21%)
Education (*N*, %)	Elementary school: 8 (21.62%)
	Middle school: 5 (13.51%)
	High school: 17 (45.94%)
	Degree: 7 (19.91%)
Job (*N*, %)	Office worker: 8 (21.62%)
	Factory worker: 4 (10.81%)
	Professional: 6 (16.21%)
	Educator/teacher: 5 (13.51%)
	Health professionals: 2 (5.4%)
	Retired: 8 (24.32%)
	Unemployed: 3 (8.1%)
Treatment (*N*, %)	Individual sessions: 29 (78.37%)
	Group sessions: 8 (21.62%)

**Table 2:** Linear mixed modeling.

	Total QUID			Sensorial			Affective			Evaluative			Mix		
	*β*	*p*	95% CI	*β*	*p*	95% CI	*β*	*p*	95% CI	*β*	*p*	95% CI	*β*	*p*	95% CI
Anxiety	1.092	0.000	0.789 to 1.396	0.400	0.000	0.264 to 0.536	0.257	0.000	0.184 to 0.331	0.231	0.000	0.137 to 0.324	0.178	0.000	0.126 to 0.229
Depression	1.051	0.000	0.621 to 1.481	0.350	0.003	0.135 to 0.566	0.228	0.004	0.116 to 0.339	0.276	0.000	0.153 to 0.400	0.181	0.000	0.115 to 0.245
Functioning	1.191	0.630	−3.714 to 6.096	0.582	0.506	−1.541 to 2.706	0.177	0.804	−1.366 to 1.720	0.814	0.223	−0.5102 to 2.139	−0.144	0.769	−1.193 to 0.904
Problems	6.158	0.000	4.661 to 6.656	2.168	0.000	1.500 to 2.837	1.537	0.001	0.951 to 2.123	1.485	0.000	0.974 to 1.996	1.027	0.000	0.714 to 1.340
Well-being	6.287	0.000	3.552 to 9.022	2.469	0.000	1.261 to 3.676	1.162	0.005	0.382 to 1.941	1.615	0.000	0.857 to 2.373	0.882	0.000	0.423 to 1.340
Risks	12.961	0.002	8.525 to 17.398	4.769	0.000	3.214 to 6.324	3.115	0.000	2.109 to 4.121	2.480	0.001	1.407 to 3.553	1.840	0.000	1.187 to 2.493

*Note*. QUID: Italian questionnaire of pain.
